# Mechanical, Thermal, and Electrical Properties of BN–Epoxy Composites Modified with Carboxyl-Terminated Butadiene Nitrile Liquid Rubber

**DOI:** 10.3390/polym11101548

**Published:** 2019-09-23

**Authors:** Xingming Bian, Rui Tuo, Wei Yang, Yiran Zhang, Qing Xie, Junwei Zha, Jun Lin, Shaojian He

**Affiliations:** 1 State Key Laboratory of Alternate Electrical Power System with Renewable Energy Sources, North China Electric Power University, Beijing 102206, China; bianxingming@ncepu.edu.cn (X.B.); pih6190001@163.com (R.T.); yr495459435@163.com (Y.Z.);; 2 State Key Laboratory of Advanced Power Transmission Technology, Global Energy Interconnection Research Institute, Beijing 102211, China; 19630100@163.com; 3 State Key Laboratory of Power System, Department of Electrical Engineering, Tsinghua University, Beijing 100084, China; zhajw@ustb.edu.cn

**Keywords:** epoxy resin, carboxyl-terminated butadiene nitrile liquid rubber, structure, mechanical properties, dielectric properties

## Abstract

Filled high thermal conductivity epoxy composite solves the problem of the low thermal conductivity of the epoxy resin itself, but the addition of the thermal conductive filler reduces the mechanical properties of the composite, which limits its application in the field of high voltage insulation. In this work, carboxyl-terminated butadiene nitrile liquid rubber (CTBN) was used to toughen the boron nitride-epoxy hybrid system, and the effects of different contents of CTBN on the mechanical properties, thermal conductivity, glass transition temperature, thermal stability, and dielectric properties of the composites were investigated. The results showed that when the content of CTBN was 5–15 wt.%, the CTBN formed a dispersed island structure in the epoxy resin matrix. The toughness of the composite increased by about 32%, the breakdown strength was improved, and the thermal conductivity was about 160% higher than that of pure epoxy resin. As the CTBN content increased, the glass transition temperature and thermal stability of the composite decreased and the dielectric constant and the dielectric loss increased. When the CTBN content is 10–15 wt.%, a toughened epoxy composite material with better comprehensive properties is obtained.

## 1. Introduction

With the development of the power grid, the demand for electrical equipment for the construction of various voltage levels of AC and DC transmission is increasing, and the amount of equipment is increasing year by year. Epoxy materials used in the electrical field are subjected to electrical, thermal, chemical, and mechanical forces in long-term operation. The energized conductor generates a large amount of heat. If at the same time, the heat generated in the insulating material is greater than the heat dissipated, the dielectric temperature will rise continuously, causing decomposition and carbonization of the insulating medium, degrading the original insulating properties. Therefore, thermal management has become an important issue in the development of epoxy materials [1,2,3,4,5].

The thermal conductivity of epoxy resin itself is very low (0.2 W/m·K), which cannot meet the actual demand. At present, the method of rapidly increasing the thermal conductivity of epoxy resin is to add a high thermal conductivity filler [6,7] such as Al_2_O_3_, AlN, BN, etc. [8,9,10]. Among them, BN is a hot spot of current research due to its good thermal stability and high thermal conductivity (320 W/m·K) [6,7,11,12,13]. However, due to the poor compatibility of BN with epoxy resin, the addition of epoxy resin causes a decrease in the mechanical properties of the composite. Insulation materials used in power equipment, such as basin insulators, saturable reactors, dry transformers, high voltage bushings, etc., have high requirements for mechanical properties, and the decrease in mechanical properties causes the composite material to rupture due to internal stress caused by temperature changes during operation, resulting in insulation breakdown [2,3].

At present, the main methods for toughening epoxy resins are the addition of inorganic nanofillers [14], thermoplastic resins [15], or rubber-based elastomers [16,17,18,19,20,21,22,23,24,25], in which the addition of liquid rubber blending into the epoxy resin system is simple and effective.

The commonly used liquid rubbers are carboxyl-terminated butadiene nitrile liquid rubber (CTBN) [16,17,18,19,20,21], epoxy-terminated butadiene nitrile liquid rubber (ETBN) [22], vinyl-terminated butadiene nitrile liquid rubber (VTBN) [23], and hydroxyl-terminated butadiene nitrile liquid rubber (HTBN) [17,24,25]. The epoxy resin and the active rubber mixed system phase separate during the curing process, and the rubber particles are precipitated from the epoxy matrix to form a sea–island structure. The dispersed rubber particles can undergo deformation to absorb energy at the time of fracture and prevent the extension of the crack, thereby achieving the purpose of toughening [19]. At present, most research is on toughening phase structure, the toughening mechanism, and the influence on the mechanical properties of toughened materials of different rubbers, but there are few studies on dielectric properties and insulation properties. The dielectric properties and insulation properties of epoxy resin insulation materials used in electrical equipment affect the stability and safety of equipment operation [26,27,28]. Therefore, it is necessary to study the changes in dielectric properties and insulation properties of rubber-toughened resin materials and to explore the feasibility of rubber for toughening of high-voltage insulation materials.

In this work, high thermal conductivity filler boron nitride (BN) was used to fill the epoxy resin to improve the thermal conductivity of the composite. At the same time, the epoxy resin was modified with CTBN. The cross-sectional morphology, mechanical properties, thermal conductivity, glass transition temperature, dielectric properties, and breakdown strength of the composites were analyzed. The effect of the addition of CTBN on the properties of epoxy boron nitride composites was investigated.

## 2. Materials

Hexagonal boron nitride (h-BN, 5–10 μm, purity of 99.0%) was purchased from Qinhuangdao Yinuo Material Co., Ltd. (Qinhuangdao, China). Epoxy resin (E-51) was supplied by Shanghai Resin Factory Co., Ltd., China. Methylcyclohexene-1,2-dicarboxylic anhydride and 2,4,6-Tris(dimethylaminomethyl)phenol were purchased from TCI (Shanghai, China) Development Co., Ltd. and used as curing agent and accelerator. Carboxyl-terminated butadiene nitrile liquid rubber (CTBN) with molecular weight of 3000 (Mn) and acrylonitrile mol content of 28% was manufactured by Tong Gao Chemical Co., Ltd. (Jingjiang, China). All materials were used as received.

### 2.1. Preparation of Composite Materials

At first, a certain amount of epoxy resin and CTBN were stirred at 60 °C for 20 min to uniformly mix the CTBN and the resin. Then, according to the mass ratio of 100:85 (epoxy:curing agent), the curing agent was added, and the amount of BN added was calculated based on 15 wt.% of the total mass of the mixed system. After stirring for 60 min at 60 °C, the mixture was treated with a high-speed dispersion machine for 15 min. After that, an appropriate amount of accelerator was added and stirred well. The mixture was then poured into a mold and degassed under vacuum at 60 °C in a vacuum oven until no significant bubbles were formed. The final mixture was cured at 100 °C for 4 h and then 150 °C for another 10 h. After cooling to room temperature and demolding, the desired sample is obtained. According to the above procedure, the epoxy resin composites with CTBN content of 0 wt.% to 30 wt.% were prepared, respectively.

### 2.2. Characterization

The tensile strength and elongation at break of the composite were measured according to the ISO 527:2012 standard using a universal testing machine (GT-TC2000, Gotech Testing Machines, Co. Ltd., China) at a speed of 10 mm min^−1^. The experimental results take the average of five samples. The unnotched impact strength of the samples was measured according to the ISO 180:2000 standard using a Zwick Roll HIT pendulum impact tester (Zwick Roll, Ulm, Germany), and 10 samples were measured per group. The above experiments were all carried out at room temperature. The fracture surfaces of the samples after the tensile tests were sputtered with gold, and the distribution of boron nitride and CTBN in the resin matrix was observed in a field emission environment scanning electron microscope Quanta 200 FEG (FEI, Hillsboro, OR, USA). The storage modulus and glass transition temperature of the samples were measured by dynamic mechanics analysis (DMA). The test was performed on a DMA Q800 (TA Instruments, Newcastle, PA, USA) instrument using a double cantilever mode from 30 °C to 200 °C at 1 Hz with the heating rate of 3 °C min^−1^. Thermogravimetric analysis was performed on TGA 5500 (TA Instruments, Newcastle, PA, USA) at a temperature ranging from 40 °C to 700 °C under a nitrogen atmosphere at a heating rate of 20 °C min^−1^. The thermal diffusivity (*α*) of the sample was measured by LFA 467 laser thermal conductivity meter (NETZSCH, Bavaria, Germany) at 25 °C, and the specific heat (*c*) of the sample was measured using a TA Q2000 differential scanning calorimeter (TA Instruments, Newcastle, PA, USA). A MH-300A density tester MH-300A (MatsuHaku, Taiwan) was used to measure the density (*ρ*) of the sample. The thermal conductivity of the sample was calculated by Equation 1, and the average of three samples in each group was taken as the thermal conductivity of the composites.

(1) k=α·ρ·c

The dielectric constant and dielectric loss of the samples were measured at room temperature using a concept 80 broadband dielectric electrical impedance analyzer (Novocontrol, Montabaur, Germany) with a sample diameter of 40 mm, a thickness of 1 mm, and a test frequency range of 10^−1^ to 10^6^ HZ. The breakdown voltage of the sample was measured by a dielectric strength tester (HCDJC-50 kV, Beijing Huace Testing Instrument Co. Ltd., China). During the test, the sample was sandwiched between the copper ball electrodes. The sample and the electrodes were immersed in the insulating oil to prevent flashover, and an increased AC voltage (50 Hz) of 2 kV/s was applied until the sample failed. Breakdown strength of 10 samples was measured per group.

## 3. Results and Discussion

### 3.1. Fracture Morphology of Composite Materials

**Figure 1:**
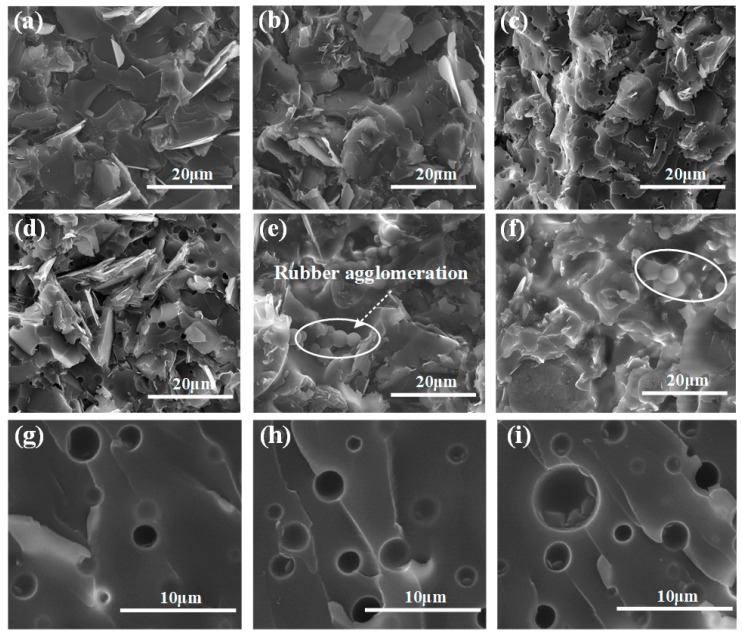
SEM images of epoxy composites. (**a**) 0 wt.% CTBN; (**b**,**g**) 5 wt.% CTBN; (**c**,**h**) 10 wt.% CTBN; (**d**,**i**) 15 wt.% CTBN; (**e**) 20 wt.% CTBN; (**f**) 30 wt.% CTBN.

Figure 1 shows the SEM images of the cross sections of the fractured samples with different CTBN content. It can be seen that the BN filler is dispersed in the resin matrix. The composite material without CTBN (Figure 1a) shows the uniform distribution of BN and relatively smooth fracture surfaces [29]. After adding CTBN (Figure 1b–d), the fracture surface is composed of two different phases, the epoxy resin is the continuous phase, and the CTBN is the dispersed phase, which is uniformly dispersed in the matrix to form an “island” structure. As the CTBN content increases, the average size of the CTBN particles increases. However, when the content of CTBN continues to increase (Figure 1e–f), as the viscosity of the system increases, the agglomeration of the CTBN causes the uniform morphology to be disturbed, which has a great influence on the toughness of the composite. Various mechanisms for rubber toughening have been reported and are believed to work alone or synergistically when toughening epoxy resins [19,30]. Figure 1g–i show the broken CTBN particles and stress whitening zone. The whitening of the stress is due to the scattering of visible light from the scattering center layer. In this case, it is the void of the scattering center, which is due to the cavitation of the CTBN particles. It is an important energy dissipation mechanism after CTBN is added into the epoxy resin, which can consume the energy at the time of fracture, and rubber bridging and shear yielding are also reasonable toughening mechanisms [31].

### 3.2. Mechanical Properties of Composites

**Figure 2:**
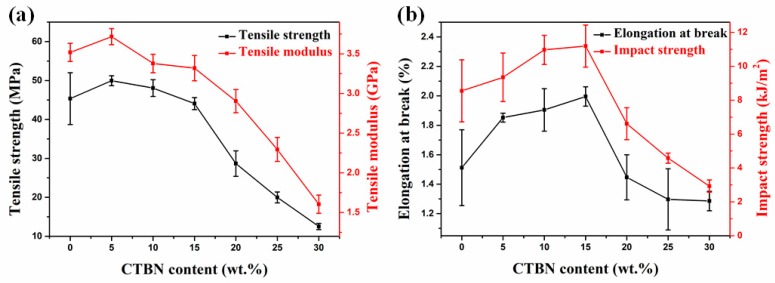
Mechanical properties of composites with different CTBN contents: (**a**) tensile strength and tensile modulus and (**b**) elongation at break and impact strength.

Figure 2 shows the variation in the mechanical properties of the composites with increasing CTBN content. When the CTBN content is 5 wt.%, the tensile strength reaches a maximum of 50 MPa (Figure 2a), which is 10% higher than that without CTBN. After that, as the CTBN content continues to increase, the tensile strength gradually decreases, but is still higher than the tensile strength when no CTBN is added, which is similar to the reported results [29,32]. The probable cause is the small CTBN particles formed by the addition of a small amount of CTBN, which fills the internal voids of the composite and increases the tensile strength of the composite as a whole [33,34], resulting in higher tensile strength than composites without CTBN addition. When the CTBN content exceeds 20 wt.%, the tensile strength of the composite material decreases significantly. On the one hand, the crosslinking density of the curing network is lowered due to the addition of CTBN. On the other hand, it can be seen from the SEM images that the CTBN particles aggregate and cannot form a dispersed structure. At this time, the CTBN is equivalent to a defect in the resin matrix and has a large influence on the tensile strength [35,36]. Similar to the behavior of the tensile strength, the tensile modulus of the composite (Figure 2b) reaches a maximum at 5 wt.% CTBN. After that, as the CTBN content increases, the tensile modulus decreases gradually and is lower than that of the composite without CTBN. The decrease in modulus is due to the addition of a low modulus rubber elastomer, and the reason for the increase in the modulus of the 5 wt.% CTBN sample is also due to the small CTBN particles filling the voids inside the epoxy resin [34].

The elongation at break increases with increasing CTBN content, reaching a maximum of 2.0% at 15 wt.%, an increase of 32%. As with the tensile strength, a significant drop occurred after the CTBN content reached 20 wt.%. The change in impact strength was consistent with the elongation at break. When the CTBN content was 15 wt.%, the maximum value of 11.2 kJ/m^2^ was observed, which was 31% higher than that without CTBN modification. The impact behavior of CTBN-modified composites can be explained based on the two-phase nature of the system [19,37]. When the crack extends to CTBN, the CTBN particles will connect the two sides of the crack like a bridge to prevent the crack from spreading to a catastrophic size; at the same time, the CTBN particles are deformed after being impacted, and the energy at the time of the fracture can be absorbed, thereby improving the toughness of the composite.

The results show that epoxy composites containing 10–15 wt.% CTBN exhibit the best balance of mechanical properties.

### 3.3. Dynamic Mechanical Properties

**Figure 3:**
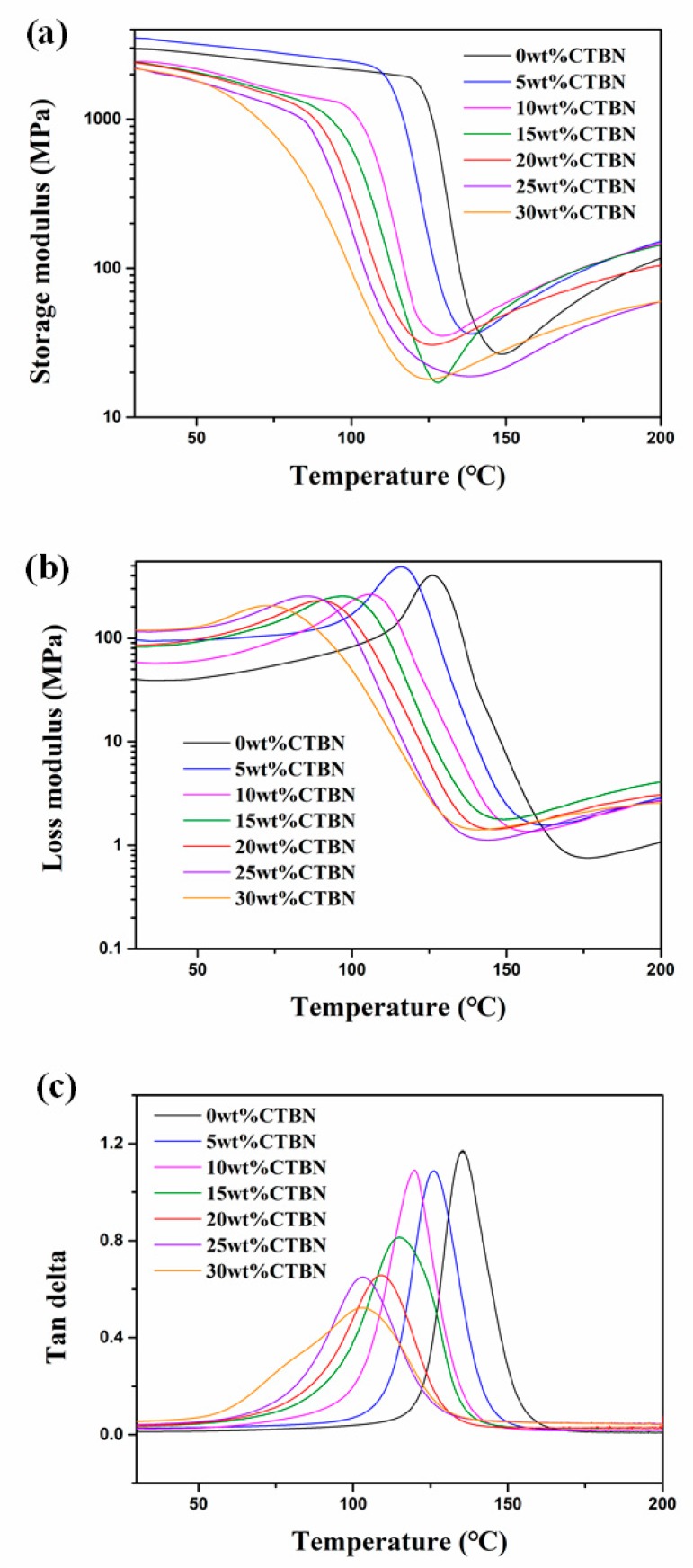
The dynamic mechanics analysis (DMA) patterns of composites with different CTBN contents: (**a**) storage modulus, (**b**) loss modulus, and (**c**) loss factor (tanδ).

Figure 3 shows the DMA test results for CTBN composites. It can be seen from the storage modulus (*E*′) of the composites with different CTBN contents shown in Figure 3a that the change of storage modulus of composites is consistent with the tensile modulus. The composite with CTBN content of 5 wt.% has the highest storage modulus, and then the storage modulus decreases with further increase of CTBN content. The loss modulus (*E*″) and loss factor (tanδ) as a function of temperature are shown in Figure 3b,c. As the CTBN content increases, the glass transition temperature (*T*_g_) of the composite material continues to decrease. Because of the good compatibility between the epoxy resin and the CTBN, part of the CTBN becomes dissolved in the epoxy matrix when the composite material is cured. The contents of the dissolved CTBN increase with the amount of added CTBN, which results in a decrease in the crosslinking density of the thermosetting network. Therefore, the number of flexible segments in the molecular chain increases, facilitating the segmental movement and lowering the *T*_g_ of the composites [19,29]. At the same time, it can be seen from Figure 3c that the full width at half maximum (FWHM) of the tanδ peak of the sample with high CTBN content is higher than that of the sample with low CTBN content, indicating that the increase of CTBN content widens the distribution of local crosslink density of the composites [29,38].

### 3.4. Thermogravimetric Analysis

**Figure 4:**
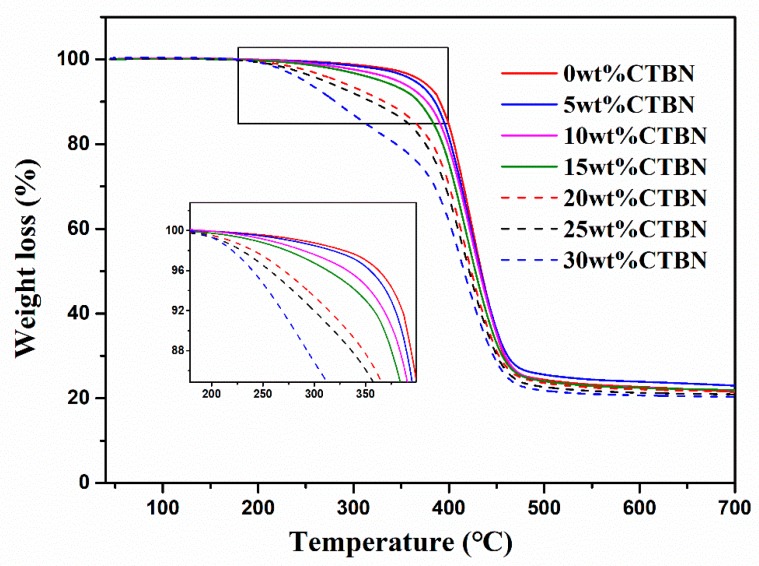
TGA curves of composites with different CTBN contents.

Figure 4 shows the thermogravimetric curves of composites with different CTBN contents. With the increase of CTBN content, the decomposition rate of the composite material is accelerated and the thermal stability is gradually reduced. Table 1 lists the temperature at which the weight loss is 10% (*T*_10%_), the temperature at which the weight loss is 50% (*T*_50%_), and the temperature corresponding to the maximum decomposition rate (*T*_max_) to compare the thermal stability of each composite. When the CTBN content is 15 wt.%, the *T*_10%_ and *T*_50%_ of the composite are reduced by about 22 °C and 7 °C, respectively, and the *T*_max_ is lowered by about 8 °C compared to those of the composite without CTBN. When the content of CTBN reaches 20–30 wt.%, the values of these temperatures are reduced much more rapidly. Especially when the CTBN content reaches 30 wt.%, the 10% weight loss temperature drops by about 109 °C, and the thermal stability is poor. The decrease in thermal stability after the addition of CTBN is due to the addition of CTBN, which reduces the crosslink density of the composite. The decrease in crosslink density leads to a decrease in the activation energy required for the degradation of the composite material, and the composite material is more susceptible to degradation, so the CTBN addition shows a decrease in thermal stability [39].

### 3.5. Thermal Conductivity of Composite Materials

**Figure 5:**
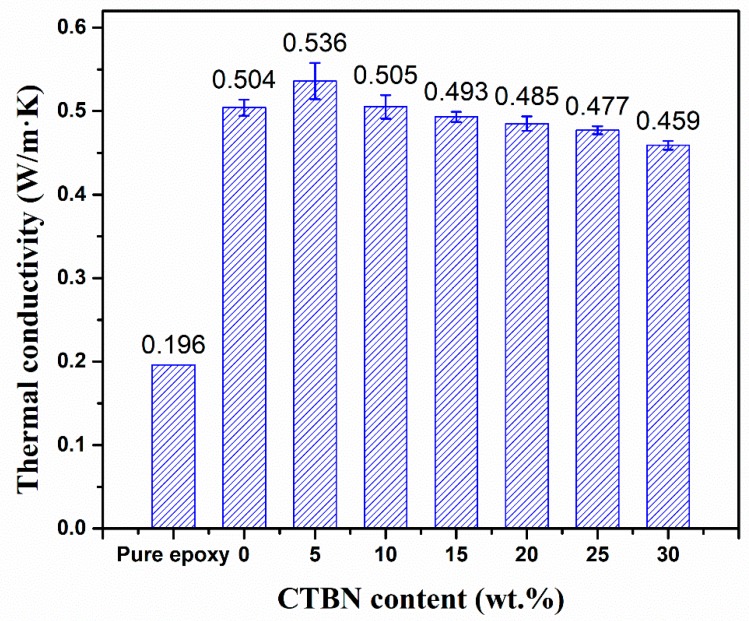
Thermal conductivity of composites with different CTBN contents.

The test results of the thermal conductivity of the composite materials are shown in Figure 5. It can be seen from the figure that the addition of high thermal conductivity BN filler is very significant for the improvement of thermal conductivity, which is about 157% higher than that of pure epoxy resin. When the CTBN content is 5 wt.%, the thermal conductivity of the sample reaches the maximum value, which is about 173% higher than that of the pure epoxy material. As the CTBN content continues to increase, the thermal conductivity of the composite material gradually decreases. The samples of 5 wt.% CTBN exhibit excellent thermal conductivity and the possible reason is that the island structure formed by CTBN dispersion can improve the internal structure of matrix after curing. For the study of highly thermally conductive materials, a large number of reports [33,40,41,42] have shown that the binary filler hybrid system exhibits a higher thermal conductivity than a single filler system, and even some nanofiber materials with low thermal conductivity can improve the thermal conductivity of composites. When the CTBN content is 5 wt.%, the particle size of the formed CTBN is small. These small CTBN particles can fill in some voids and defects due to the addition of BN and autocuring of the epoxy resin, such that the composite material has a higher thermal conductivity [33]. When the CTBN content is above 10% by weight, the effect of filling voids and defects is weakened due to the increase of the particle size of the CTBN particles, and the thermal conductivity of the CTBN itself is lower than that of the epoxy resin, resulting in a gradual decrease in the thermal conductivity of the composite.

### 3.6. Dielectric Properties

**Figure 6:**
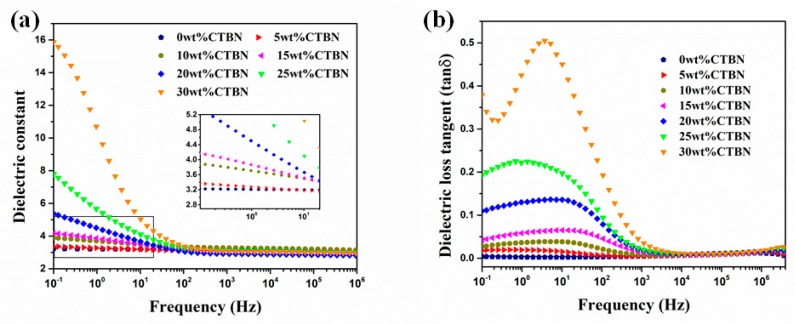
Dependence of (**a**) the dielectric constant and (**b**) the dielectric loss on frequency for composites with different CTBN contents.

The relationship between the dielectric properties of the composite and the frequency is shown in Figure 6. As can be seen from Figure 6a, as the frequency increases, the dielectric constant of the composite exhibits a downward trend. This is because dipole polarization and interfacial polarization cannot keep up with changes in the electric field frequency as the frequency increases, resulting in a decrease in dielectric constant [43]. In the high frequency region (10^2^–10^6^ Hz), the dielectric constants of the composite materials are not much different, and the difference is mainly reflected in the low frequency region (10^−1^–10^2^ Hz). When the frequency is low, the dielectric constant of the composite increases with the increase of CTBN content. This is because the addition of CTBN creates a new resin–rubber interface inside the composite. Due to the difference in polarity and conductivity between CTBN and resin, electrons and ions will accumulate at the interface under the action of an external electric field, resulting in interfacial polarization, so the dielectric constant of the composite increases [44]. In particular, after the CTBN content reaches 20 wt.%, the CTBN aggregates are no longer well dispersed in the resin matrix, and the interfacial polarization effect is more pronounced, resulting in a significant increase in the dielectric constant in the low frequency region. In addition, due to the addition of CTBN, the crosslink density of epoxy resin is reduced, and more side chains and branches are present inside the matrix, which is also the reason for the increase of dielectric constant of composite materials. With the addition of CTBN, the dielectric loss tangent of the composite increases (Figure 6b), which is also due to interfacial polarization and the decrease of crosslink density. In the low frequency region, the dielectric loss of the composite first increases and then decreases, which is due to the influence of dielectric relaxation [43,44]. In the high frequency region, as the frequency increases, the dielectric loss increases slightly, which is caused by the dielectric properties of the epoxy resin itself, because the interface polarization is not obvious at high frequencies [35].

### 3.7. Breakdown Strength

Breakdown strength is an important property of composite materials and the experimental data for breakdown strength is typically processed using the Weibull function. The two-parameter Weibull formula is

(2) $$F\left(x\right)=1-\exp\left(-\frac{x^{\beta }}{\alpha }\right)$$

where *x* is the breakdown strength, the unit is kV·mm^−1^, *α* is the proportional parameter, the unit is kV·mm^−1^, and *β* is the shape parameter, which is the slope of the fitted curve. 

**Figure 7:**
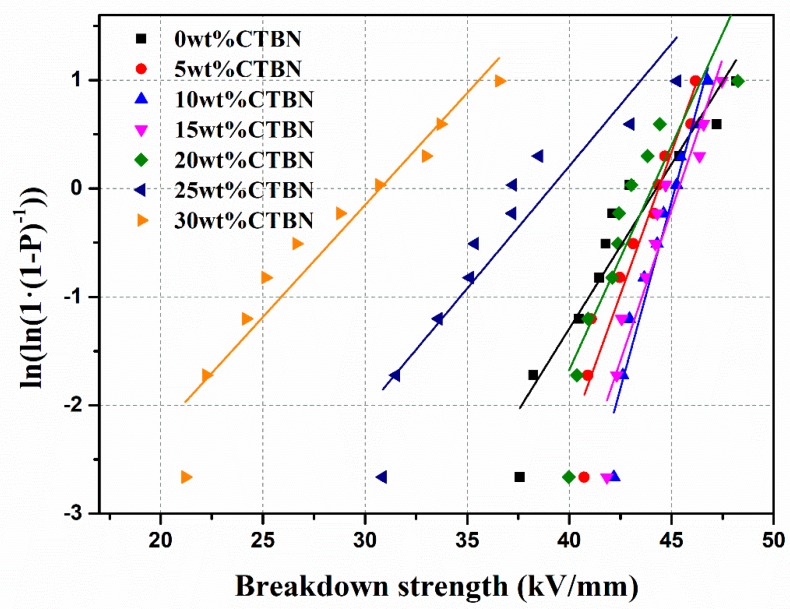
Weibull distribution of composite material breakdown strength.

The test results of the breakdown strength of composites with different CTBN contents are shown in Figure 7 and Table 2. The results show that as the CTBN content increases from 0 wt.% to 15 wt.%, the breakdown strength of the composite increases from 44.1 kV to 45.3 kV, the small addition of CTBN has a certain improvement effect on the breakdown strength of the composite. The possible reason is that the short-time breakdown voltage is developed in the form of electric branches [27]. When the electric branches extend to BN, the electric branches will continue to extend along the interface between the BN filler and the epoxy resin. This part of the interface has a low breakdown strength, and the electric branches develop very fast here. The addition of CTBN could affect the spatial distribution of BN, which reduces the interfacial void between BN and the resin, as CTBN can form a weak cross-linking reaction with the resin due to its own carboxyl group, which can fill the defects generated in the curing process of the resin [34]. Compared with the interface between BN and epoxy resin, due to the weak cross-linking reaction, the interface between CTBN and resin has a stronger ability to inhibit the growth of electric branches. The shape parameter *β* reflects the reflects the dispersion of data. It can be seen from the table that when the amount of CTBN added is 5–15 wt.%, the shape parameter is significantly higher than that of the resin without CTBN added, which also reflects from the side that the uniformity of the interior of the composite is improved after the addition of CTBN. As the CTBN continues to be added, the CTBN agglomerates and introduces more defects, such that the breakdown strength of the composite material is rapidly reduced.

## 4. Conclusions

In this work, a carboxyl-terminated butadiene nitrile liquid rubber (CTBN) was used to toughen the BN–epoxy resin composite system, and the mechanical, thermal, and dielectric properties of the composites were studied. The conclusions are as follows:

(1) When the amount of CTBN added was 5–15 wt.%, a dispersed sea–island structure was formed after the curing system was solidified, and the toughness of the composite material was improved. The impact strength and elongation at break of the composite containing 15 wt.% CTBN were both increased by about 32%, and the tensile strength was only slightly decreased.
(2) The addition of CTBN reduced the crosslink density of the epoxy resin, the glass transition temperature of the composite decreased, and the thermal stability decreased. However, when the content of CTBN was low (5–15 wt.%), the decrease in thermal performance was within an acceptable range.
(3) The composite containing 5 wt.% CTBN had the highest thermal conductivity, which was about 173% higher than that of pure epoxy resin. The dielectric constant of the composite increased with the addition of CTBN, and a peak appeared in the dielectric loss curve due to the influence of dielectric relaxation. At the same time, the addition of CTBN had a certain improvement effect on the breakdown strength of the composite.
(4) The composite material with 10–15 wt.% CTBN content showed the best performance balance, good toughness, high glass transition temperature and thermal stability, high breakdown strength, but in practical application, the working conditions of high-voltage equipment should be fully considered to avoid excessive dielectric loss caused by dielectric relaxation.

## Figures and Tables

**Table 1:** Thermal stability of CTBN epoxy resin composites.

Sample	*T*_10%_ (°C)	*T*_50%_ (°C)	*T*_max_ (°C)
0 wt.%CTBN	390	434	425
5 wt.%CTBN	385	433	422
10 wt.%CTBN	377	432	423
15 wt.%CTBN	368	427	417
20 wt.%CTBN	332	423	416
25 wt.%CTBN	319	421	417
30 wt.%CTBN	281	415	414

**Table 2:** Weibull distribution parameters of breakdown strength of samples.

Sample	Scale Parameter *α* (kV/mm)	Shape Parameter *β*
0 wt.% CTBN	44.1	13.1
5 wt.% CTBN	44.4	22.8
10 wt.% CTBN	45.1	30.7
15 wt.% CTBN	45.3	24.0
20 wt.% CTBN	44.0	18.5
25 wt.% CTBN	39.7	6.4
30 wt.% CTBN	29.4	5.8
